# Prebiotics Enhance Microbiome Recovery Following Antibiotic-Induced Dysbiosis

**DOI:** 10.3390/microorganisms14051079

**Published:** 2026-05-11

**Authors:** Paige Ramkissoon, Anthony DuCasse, Isaac Berman, Jonathan Sadanaga, Ian O’Neill, David C. Gondek

**Affiliations:** Biology Department, Center for Natural Science, Ithaca College, Ithaca, NY 14850, USA

**Keywords:** microbiome, antibiotic resistance, prebiotic, probiotic, short-chain fatty acids

## Abstract

Antibiotic-induced dysbiosis disrupts gut microbiome diversity and functionality, often leading to negative health outcomes, including reduced short-chain fatty acid production, increased susceptibility to opportunistic pathogens, and an increased number of bacterial colonies exhibiting antibiotic resistance. This study investigates the effects of prebiotics (inulin-type fructans) and probiotic supplementation on microbiome recovery in a murine model. Broad spectrum antibiotics induced near-total microbiome depletion, significantly reducing microbial diversity and metabolite production. Prebiotic supplementation demonstrated superior efficacy during recovery in restoring microbiome diversity (~180 species), improving microbiome diversity metrics, and promoting metabolites, particularly butyrate and valerate, compared to probiotics or unmanipulated recovery. While effective in suppressing opportunistic bacterial growth, probiotics significantly delayed total microbial diversity recovery and resulted in lower diversity metrics (~50 species). However, prebiotic-treated microbiomes exhibited a wider antibiotic resistance profile in culturable bacteria, highlighting prebiotics’ unique impact on the resistome. These findings underscore the potential of prebiotics for recovery from gut dysbiosis while emphasizing the need for further research to address safety considerations regarding their impacts on antibiotic resistance. Importance: This study explores the impact of prebiotic vs. probiotic manipulation of the microbiome in an antibiotic-induced dysbiosis mouse model. Our data demonstrate that prebiotics are more efficacious at enhancing total diversity and limiting the expansion of potentially harmful opportunist bacteria. This is the first study to indicate that prebiotics increase the number of culturable bacterial colonies resistant to antibiotics. These results contribute to our understanding of microbiome manipulation to promote health and limit disease.

## 1. Introduction

The role of the gut microbiome in human health is increasingly recognized, with research highlighting the impact of its composition and function on various physiological processes. The development and application of antibiotics have significantly contributed to public health by combating bacterial infections [1,2,3] and improving the safety of elective surgeries [4]. However, antibiotic use can disrupt this delicate microbiome balance, leading to negative consequences, including antibiotic-associated diarrhea (AAD), an increased risk of pathogen colonization, obesity, diabetes, and autoimmune disorders [5,6,7,8,9]. Dysbiosis, as defined by Ley et al., refers to a bloom of opportunists, loss of commensals, or loss of diversity [10]. This highlights the critical role of a balanced gut microbiome in maintaining overall health and preventing disease.

Moreover, many bacterial taxa have evolved antimicrobial resistance (AMR) to several classes of antibiotics, and multidrug-resistant bacteria prevent the comprehensive treatment of infections. The overall resistome can expand following these selection events and/or expand due to the introduction of new bacteria to the microbiome, such as opportunistic colonizers or via transfer from probiotics [11,12]. Indeed, many labs have examined the role of probiotics in the treatment of antibiotic-induced diarrheal disease and may have contributed to this expansion of AMR [3,9,13].

Recent studies have demonstrated that prebiotic diets can regulate the composition of the host’s intestinal microbial community and mitigate the development of diseases caused by microbial imbalances [14,15]. In particular, inulin-type fructans, which are rich in polyfructosan soluble dietary fibers, are recognized as effective prebiotics [16,17]. Natural sources of inulin include chicory roots, Jerusalem artichoke, dahlia tubers, garlic, and leeks. Inulin prebiotics, which vary in their degree of polymerization, play significant roles in improving intestinal microecology, promoting fatty acid metabolism [18,19], and enhancing the absorption of ions and minerals in the intestinal tract [20].

One of the primary effects of prebiotics on the gut is their breakdown into short-chain fatty acids (SCFAs) by the microbiome [18]. The gut microbiome produces SCFAs, primarily acetic, propionic, butyric, and valeric acids, which serve as energy sources for colonocytes and substrates for hepato-metabolic pathways [21,22,23]. SCFAs also function as signaling molecules, binding to receptors on enteroendocrine cells to increase the secretion of gut hormones and improve blood glucose regulation [24]. Increased production of SCFAs, especially butyric acid, is considered beneficial. Studies have shown that individuals with autoimmune conditions, such as type 2 diabetes, have lower microbial diversity, fewer butyrate-producing bacteria, and lower fecal concentrations of SCFAs compared to healthy individuals [25,26]. Enhancing SCFA production through dietary interventions, such as prebiotics like inulin, could improve individuals’ metabolic outcomes.

While many studies have investigated the effects of probiotics or prebiotics on the gut microbiome following antibiotic use, rarely are they directly compared. This study seeks to fill this gap in the literature by evaluating the SCFAs, cecal morphology, alpha diversity, beta diversity, taxonomic composition, and AMR of antibiotic-treated mice that receive probiotics, prebiotics, or no additional supplements during recovery from dysbiosis. We hypothesized that prebiotic treatment would be most effective in re-establishing microbiome diversity. By examining the interplay between prebiotics and the gut microbiome, this research seeks to elucidate how to support microbiome recovery and enhance microbial diversity following antibiotic treatment.

## 2. Methods

### 2.1. Animals and Treatment Groups

**Figure 1:**
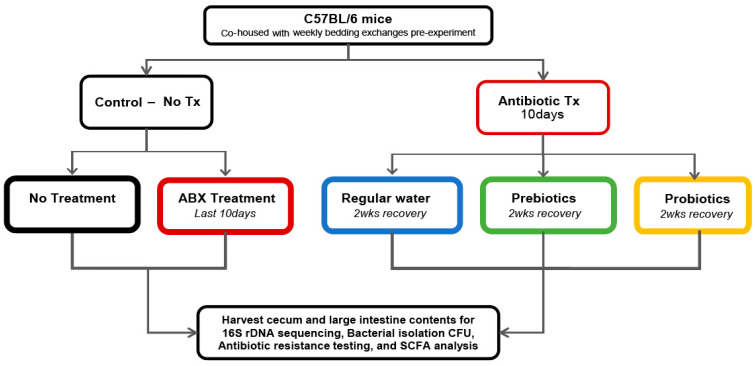
A flow chart of the experimental design. The number of animals in each treatment group were: NoTx (n = 16), water (n = 15), prebiotics (n = 15), probiotics (n = 12), and ABX treatment (last 10 days) (n = 7). Samples were collected in three biological replicates (two replicates for ABX treatment).

C57BL/6 female mice, 5–7 weeks, were obtained from Jackson Laboratory (Bar Harbor ME) at six to eight weeks of age and housed in the Ithaca College animal facility. Only female mice are used by our lab group as some animals serve as “negative controls” for other female reproductive tract microbiome studies as well. Groups of the animals were co-housed, at four to five mice per cage, with mixing of bedding between cages for three to four weeks prior to the start of antibiotic treatment. Once the experiment began, we minimized any cross contamination between the Tecniplast microisolator cages. Ampicillin (Abraxis Pharma), streptomycin (Abraxis Pharma), and clindamycin (Pharmacia) were provided into sterile drinking water at a final concentration of 1 mg/mL, as previously described for two weeks [3,27]. These antibiotics were chosen due to their broad spectrum capacity and well-documented impacts on intestinal microbiota [28]. The animals were allowed to *drink ad libitum* for the duration of the experiment with water replacement at three-day intervals. A probiotic blend of *Lactobacillus* and *Bifidobacterium* (CVS Maximum Strength Probiotic) was resuspended in five mls PBS for a total of 4 billion organisms per milliliter. The probiotics were provided to the mice via oral gavage (0.1 mL/mouse) every other day for two weeks. Dried mouse food pellets were also soaked in the remaining probiotic suspension and provided to the mice. Prebiotic Inulin Fiber-FOS (Jarrow) was mixed with 0.3 g/mL of drinking water. The control groups were left unmanipulated for the duration of the study (No Tx) or treated with antibiotics for the last ten days before analysis (ABX) (Figure 1). When our lab shifted from open air to microisolator cages, we noted the substantial change in microbiome dynamics and the data presented here only represent experiments using microisolator cages. Opportunistic pathogen differences between open air and microisolator cage setups are noted in Table 1. The Ithaca College Institutional Animal Care and Use Committee approved all experiments and protocols.

### 2.2. Sample Collection and SCFA Analysis

Following CO^2^ asphyxiation, fecal matter was extracted from individual mouse cecum and large intestine, snap frozen, and later derivatized using a pyridine/propanol protocol previously described [29]. Briefly, the samples were thawed by suspension in 0.005 M NaOH, homogenized, and centrifuged. Added to each sample was 500 mL Propanol/Pyridine (3:2) and 100 mL Propylcholoformate, which was sonicated for one minute. After derivatization, a two-step hexane extraction was used to isolate the SCFAs. The amount of each SCFA was analyzed using gas chromatography–mass spectrometry (Shimadzu GCMS-QP2010, Kyoto, Japan) and normalized to spiked internal standard 4-methyl-valerate (500 µM).

### 2.3. DNA Extraction and Sequencing

DNA was extracted and analyzed from fecal bacteria using the PowerFecal Pro (Qiagen, Valencia, CA, USA) DNA isolation protocol. The 16S rRNA gene V4 variable region PCR primers 515/806 were used in a 30-cycle PCR using the HotStarTaq Plus Master Mix Kit (Qiagen, Valencia, CA, USA) under the following conditions: 95 °C for 5 min, followed by 30–35 cycles of 95 °C for 30 s, 53 °C for 40 s and 72 °C for 1 min, after which a final elongation step at 72 °C for 10 min was performed. After amplification, the PCR products were checked in 2% agarose gel to determine the success of amplification and the relative intensity of bands. The samples were multiplexed using unique dual indices and were pooled together in equal proportions based on their molecular weight and DNA concentrations. The pooled samples were purified using calibrated Ampure XP beads(Beckman Coulter, Indianapolis, IN, USA). Then the pooled and purified PCR product was used to prepare an Illumina DNA library. Sequencing was performed at MR DNA (www.mrdnalab.com, Shallowater, TX, USA) on a MiSeq following the manufacturer’s guidelines. The sequence data were processed using MR DNA analysis pipeline (MR DNA, Shallowater, TX, USA). In summary, the sequences were joined, sequences < 150 bp removed, and sequences with ambiguous base calls removed. The sequences were quality-filtered using a maximum expected error threshold of 1.0 and dereplicated. The dereplicated or unique sequences were denoised; unique sequences were identified with sequencing and/or PCR point errors and removed, followed by chimera removal, thereby providing a denoised sequence or zOTU. Final zOTUs were taxonomically classified using BLASTn (v2.17.0) against a curated database derived from NCBI (www.ncbi.nlm.nih.gov). The data are available at Biosample SUB16101131 (NCBI).

We applied the “Quantitative Insights Into Microbial Ecology” (QIIME2) software as part of our analysis. To eliminate the differences in sequencing effort between samples, α diversity was calculated using the Shannon index and Faith-PD index based on 77,000 reads per individual. To examine β diversity, unweighted UniFrac PcoA biplots were produced. The datasets are deposited in the NCBI sequence read archive for public access.

### 2.4. Total CFU and Antibiotic Resistance

The fecal samples were diluted in Trypticase soy broth and plated on TSA plates for total bacterial count enumeration. The samples were plated in triplicate for anaerobic, microaerophilic, and aerobic growth conditions. Based on visual colony characterization, forty unique colonies per treatment group were collected, streaked to isolation, and further analyzed. The antibiotic resistance of the fecal bacteria was measured using the Kirby–Bauer disk diffusion susceptibility test protocol. Isolated fecal colonies were cultured on trypticase soy agar along with six antibiotic disks: penicillin, ampicillin, streptomycin, gentamicin, chloramphenicol, and clindamycin for twenty-four hours in microaerophilic conditions.

### 2.5. Statistics

The data presented are inclusive of three separate biological replicate experiments, except where noted and an experiment is a representative example. The number of animals in each treatment group were: NoTx (n = 16), water (n = 15), prebiotics (n = 15), probiotics (n = 12), and ABX (last 10 days) (n = 7). All treatment groups can be differentiated when the taxonomic data are included in the QIIME diversity analysis. The Shannon indices, Faith-PD, cecal morphology, and SCFAs were each compared among treatment groups and were examined for normality using the Shapiro–Wilk Test and a one-way Kruskal–Wallis test. In all cases we conducted a posthoc Dunn’s test to identify pairwise differences. Beta diversity generated principal coordinate analysis (PCoA) plots, which were assessed by permutation multivariate analysis of variance (PERMANOVA) in QIIME2. Relative differential abundance was assessed at the genus level using an analysis of the composition of the microbiomes (ANCOM-BC) in QIIME2. Figures were created in GraphPad PRISM (v11.0.0) (* = *p* < 0.05, ** = *p* < 0.01, *** = *p* < 0.005, **** = *p* < 0.001).

## 3. Results

**Figure 2:**
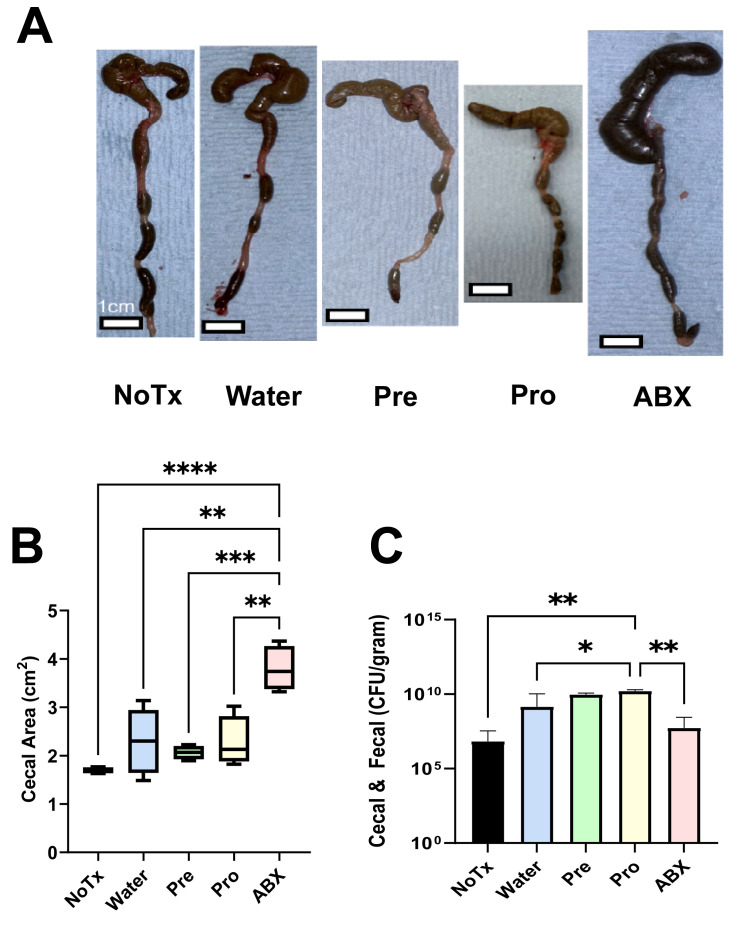
Cecal distention due to antibiotic treatment and subsequent recovery. (**A**) Representative cecal images align with the treatment group area calculations. Bar = 1 cm. (**B**) The colony-forming units per gram of stool samples (cecal + fecal). (**C**) Calculation of culturable bacteria from cecal and fecal contents within each treatment group (CFU/gram). The colored bars are all treated with antibiotics and microbiomes were supplemented, as described on the X-axis, during the two weeks of recovery. The averages represent serial dilution plates incubated in aerobic, anaerobic, and microaerophilic conditions of a single representative biological replicate (* = *p* < 0.05, ** = *p* < 0.01, *** = *p* < 0.005, **** = *p* < 0.001).

To discover the impact of prebiotics and probiotics on microbiome recovery, we isolated microbiome contents from mice forced into dysbiosis by depleting their microbiomes with broad spectrum antibiotics, after which we manipulated their microbiome diversity recovery (Figure 1). We hypothesized that the supplementation of prebiotics and probiotics would enhance microbiome diversity relative to an unmanipulated recovery process. Antibiotic treatment caused near-total microbiome depletion, significantly enlarging the cecum, alerting its color, and changing its texture (Figure 2A). Meanwhile, all animals allowed to recover from antibiotic treatment exhibited cecums that had returned to normal size. When microbial contents were plated onto agar plates for colony-forming unit (CFU) enumeration, we found a trend that all dysbiotic mice harbored a greater number of culturable bacteria compared to the untreated negative control (Figure 2B, *p* < 0.05). Probiotic-treated animals had the highest culturable bacteria load (1 × 10^10^ CFU/Gram), but this was not statistically significant relative to prebiotic-treated mice.

**Figure 3:**
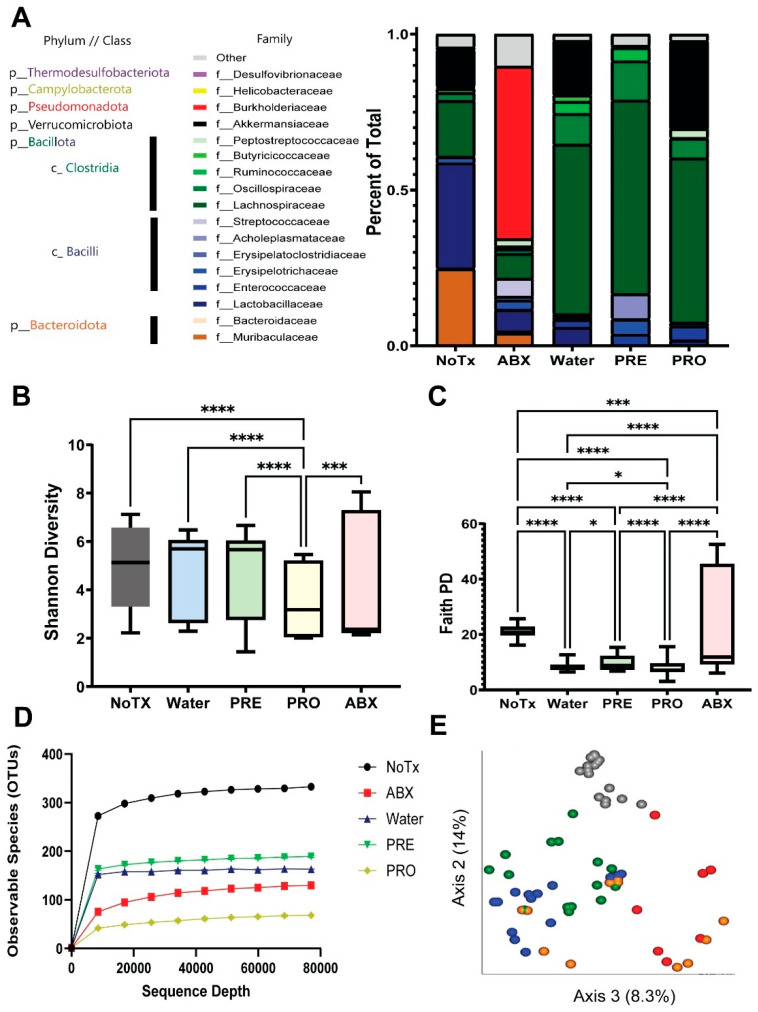
Prebiotics enhance the taxonomic composition of the mouse microbiota following recovery from antibiotic-induced dysbiosis. (**A**) Abundance of bacteria by taxonomic similarity. Alteration of alpha and beta diversity in microbiome samples: (**B**) Shannon Diversity, (**C**) Faith-PD Diversity, (**D**) Rarefaction curves, (**E**) unweighted UniFrac analysis (* = *p* < 0.05, *** = *p* < 0.005, **** = *p* < 0.001) (the data presented includes all three biological replicates).

**Figure 4:**
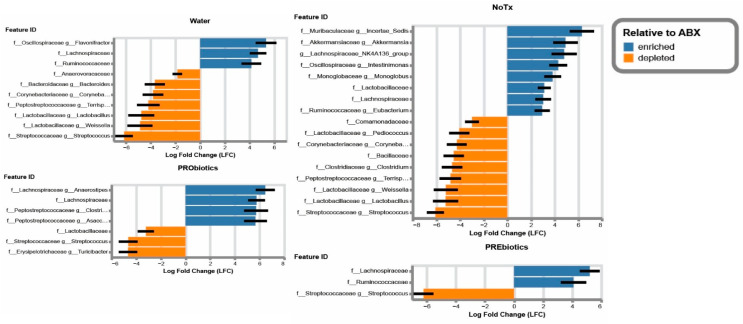
Microbiome recovery from antibiotics results in changes among the relative abundances of several taxa based on an ANCOM-BC analysis. The data are mean ± SEM. All the taxonomic families listed are statistically significant (*p* < 0.001). The genus is provided where possible. Representative of three biological replicates.

Shifts in microbiome abundance and diversity were assessed by 16S genomic sequencing of the microbiome. Unmanipulated mice had a diverse microbiome consisting of over 300 unique species, such that ~80% of the microbiome consisted of *Bacteroidota* (20%) and *Bacillota* (60%) with many family-level phylogenetic groups represented by a single family (Figure 3A). Upon antibiotic depletion for ten days, we found significant suppression of that diversity, such that 60% of the microbiome consisted of *Beta-proteobacteria* of the *Burkholderia* genus. When antibiotic treatment was stopped, and the microbiomes were allowed to recover, we sought to manipulate that recovery process with the application of prebiotics (Inulin-FOS) or a commercially available suite of probiotics (*Lactobacilli* sp. and *Bifidobacterium* sp.). We found that in all cases of microbiome recovery, *Bacteroidota* failed to recolonize in the two-week recovery period, leading to a microbiome consisting almost entirely of *Bacillota*. *Lachnospiracea* were the most abundantly detected bacteria in the recovered microbiomes, consisting of greater than 50% of the microbiome. Similarly, *Acutalibacter* and *Flavoniftactor* (family *Oscillospiraceae*) expanded in an unmanipulated recovery but were suppressed by adding prebiotics or probiotics to this recovery process (Figure 4). *Akkermansiaceae* were found in the recovered microbiomes of probiotic and water treatment, but prebiotic supplementation suppressed *Akkermansia* establishment in the microbiome. Probiotics created a niche for *Peptostreptococcacea,* such as *Clostridia,* to become highly abundant in the microbiome (Figure 3A and Figure 4). Antibiotic-treated mice had an abundance of opportunistic pathogens, such as *Burkholderia*, *Streptococcus*, *Clostridium*, and *Corynebacterium*. Notably, in the midst of doing these experiments we shifted from open air cages to HEPA-filtered microisolator cages, and this caused a dramatic shift in the types of opportunist pathogenic bacteria. In open air cages, the recovering microbiomes consisted of the *Enterobacteriaceae* family (*Escherichia*, *Shigella*, *Klebsiella*, etc.). In contrast, when we switched to sealed microisolator cages, the microbiomes had non-pathogenic bacteria (such as *Anerostripes*, *Acutalibacter*, *Bacteroides*, *Blautia*, etc.) (Figure 1).

We found that prebiotics were most impactful at establishing microbiome diversity as measured by alpha and beta diversity. Upon recovery from dysbiosis, adding prebiotics helped expand that diversity to ~180 unique species and a statistically more diverse collection than probiotic treatment, as measured by Shannon Diversity (Figure 3B). In contrast, the probiotics appeared to suppress species diversity (~60) compared to unmanipulated water recovery, without supplementation (~150 unique species) (*p* < 0.001 Figure 3B,D). Moreover, the prebiotic treatment was better at re-establishing beta diversity within the microbiome with some animals approaching a species beta diversity most similar to the unmanipulated mice (Figure 3E). In contrast, the beta diversity analysis of probiotic treatments demonstrates a “lag,” and some probiotic-treated animals with a more similar microbiome to the antibiotic-treated animals. The PERMANOVA analysis of antibiotic (Abx) and non-treated (NoTx) groups were unique groups (*p* < 0.001), whereas recovery populations given just water, prebiotics, or probiotics were more intermediate but each unique beta diversity populations (*p* < 0.05). Collectively, prebiotic supplementation during recovery from dysbiosis brings the host animals most closely back to the pretreatment microbiome based on beta diversity analysis, albeit missing an entire phylum of Bacteroidota.

**Figure 5:**
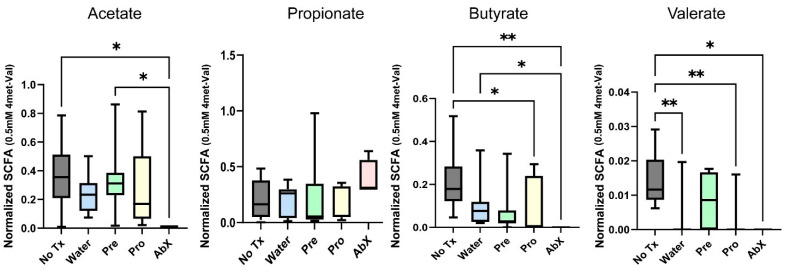
Prebiotics augment short-chain fatty acid production by the recovering microbiome (* = *p* < 0.05, ** = *p* < 0.01). Representative sample of three biological replicates.

The production of SCFAs by the microbiome is an excellent indicator of microbiome recovery, as robust SCFA production is associated with microbiome diversity. Upon depletion of the microbiome by antibiotics, we found a complete loss of butyrate and valerate production and a sharp decrease in Acetate production relative to the untreated control mice (Figure 5). The cessation of the antibiotics and recolonization resulted in a return of normal Acetate production, but butyrate production lagged significantly. Valerate production only returned to “normal” levels in the mice provided with prebiotics during their microbiome recovery process.

**Figure 6:**
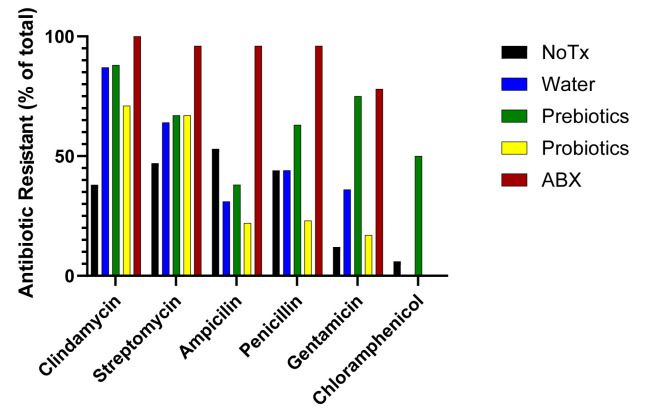
Prebiotics and probiotics alter the antibiotic resistome of culturable bacteria. Changes in the culturable bacteria antibiotic resistome occurs for both the antibiotics used in the treatment regime (clindamycin, streptomycin, and ampicillin) and antibiotics not used in the treatment (penicillin, gentamicin, and chloramphenicol). (n = 40 unique colonies per condition, representative of three biological replicates).

Following an antibiotic depletion event, we hypothesized that we would see more antibiotic-resistant bacterial colonies in treated samples. Picking from the CFU/gram plates (Figure 2B), forty unique bacterial colonies were cultivated from each experimental treatment condition. Each cultivated colony was streaked to isolation before being tested for its antibiotic resistance profile. We found that non-manipulated animals harbored a potential resistome to all antibiotic types tested, with the least resistance to gentamicin and chloramphenicol (Figure 6, black bars). For the positive control group, which was in the midst of antibiotic treatment, we found near-total resistance to the three antibiotics used (clindamycin, streptomycin, and ampicillin), as well as resistance to penicillin and gentamicin but not chloramphenicol (Figure 6, red bars). In the experimental groups where the mice were allowed to recover from the dysbiotic-inducing event, the resistome profile was unique from the two controls with intermediate resistance to ribosomal inhibitors (clindamycin and streptomycin) but exhibited a considerable drop in bacterial colony resistance to peptidoglycan inhibitors (ampicillin and penicillin). Intriguingly, the prebiotic-treated mice deviated from all other recovery groups, with many bacterial colonies demonstrating continued bacterial colony resistance to penicillin, gentamicin, and chloramphenicol (Figure 5, green bars).

## 4. Discussion

This study investigates the impacts of prebiotics (inulin-type fructans) and probiotic supplementation on gut microbiome recovery following antibiotic-induced dysbiosis in mice. Antibiotics caused near-total microbiome depletion, reducing microbial diversity and increasing susceptibility to harboring AMR microbes. Recovery treatments were compared via microbiome sequencing, CFU enumeration, and SCFA production analysis. The prebiotics demonstrated superior effectiveness in restoring microbiome diversity than the probiotics or unmanipulated recovery. Notably, the prebiotics and probiotics exhibited unique profiles across multiple metrics, emphasizing their distinct impact. These findings highlight the potential to manipulate microbiome diversity following antibiotic disruption.

Our study highlights several key points. First, prebiotics and probiotics effectively inhibit the outgrowth of opportunistic bacteria following an antibiotic-induced microbiome dysbiosis. This is particularly important in the context of bacteria implicated in antibiotic-associated diarrheal disease (*Escherichia*, *Salmonella*, and *Klebsiella*) [8]. With the switch to microisolator cages, we saw an expansion of non-pathogenic bacteria in the majority, such as *Anerostripes*, *Bacteroides* and *Acutalibacter*. These bacteria are associated with beneficial microbiome effects, although *Acutalibacter* has also been identified as a proinflammatory biomarker for both cancer and post-stroke dysphagia [30,31]. In contrast, prebiotics and probiotics treatment did not affect the colonization of other biomarker organisms, such as *Fusimonas*, a bacteria associated with metabolic disorders [32,33]. In agreement with Zmora et al., we found that treatment with probiotics suppressed the overall diversity of a repopulating microbiome without the probiotics themselves colonizing the gut [34]. In contrast to Alp and Kuleasan, we found that the probiotics did exert a remodeling effect on antibiotic-treated recovering mice, although their lack of mucosal adhesion made the probiotic bacteria only transient residents [35]. In a fully competent microbiome, the lack of probiotic adhesion combined with commensal microbe colonization resistance inhibits any effect of the probiotic, whereas germ-free mice were easily remodeled by a probiotic [34]. We demonstrate that the antibiotic recovery window provides an intermediate result, wherein transient colonization provides the opportunity to influence remodeling, but as the microbiome fully recovers the probiotics are outcompeted. This transient window has significant implications for interpreting probiotic efficacy. Second, our experience with both open air and microisolator cages highlights some of the advantages of working with ‘dirty mice,’ and that specific pathogen-free mice do not well replicate human microbiome and immune system traits [36,37,38]. To characterize these changes we found the beta diversity analysis of the 16S genomic sequencing to be the most useful approach. Collectively, we found that prebiotics and probiotics can reshape the microbiome and decrease the abundance of particular opportunistic bacteria during the microbiome recolonization process.

A second key point from our study is the effects of prebiotics and probiotics on the mouse metabolome, specifically the production of SCFAs. High microbial diversity is associated with higher levels of SCFAs, and these bacterial lipid metabolites are biomarkers of improved host organism health [39]. Butyrate and valerate are beneficial because they maintain gut barrier function, reduce inflammation, and improve glucose homeostasis [18,21]. Butyrate can induce the release of GLP1, implicating the role of the microbiome in blood sugar and weight control [40]. The relative abundance of different SCFA-producing bacteria, such as *Ruminococcus*, *Clostridium*, *Lachnoclostridium*, and *Eubacterium*, can vary and impact the overall SCFA profile [41]. Similarly, our data indicate a *Eubacterium* expansion in the prebiotic-treated animals is associated with increased valerate production in the gut [42]. Therefore, enhancing SCFA production, especially butyrate and valerate, through dietary interventions could improve host organism metabolic outcomes.

The third key point from our study is the impact of prebiotics and probiotics on the number of culturable AMR bacteria after recolonization from an antibiotic-induced dysbiosis. We found that an unmanipulated microbiome already harbored a collection of culturable bacteria resistant to nearly all the antibiotics tested [1]. We hypothesized that mice treated with probiotics would have the highest resistance metrics following recolonization, as previous studies have shown that probiotic treatment can contribute new sources of AMR genes to the microbiome [11,12]. As such, a careful analysis of probiotics is a necessary part of safety profiles for their use [13]. However, the prebiotics exhibited the highest number and widest range of AMR culturable bacteria in our study. In particular, there was an enrichment on the agar plates for an alpha hemolytic, Gram-positive coccus. Notably, the prebiotic treatment had enriched for *Enterococacea* which is also evident in the genomic abundance analysis (Figure 3A). *Eneterococcus* species are known to exhibit multidrug resistance [43]. Given the inherent cultivation bias of selecting bacteria which grow on agar plates, long detection time, and limited colony-based workflow, a more holistic genomic approach will provide a clearer understanding of the impact prebiotics have on AMR cultivation within the microbiome.

Despite the high repeatability of overall trends, our study has several limitations. As is common with microbiome work, experiment-to-experiment variation leads to different microbiome profiles for each experimental cohort and comparisons to an untreated cohort may be an inadequate control. We do see that *Bacteroidota* never return in our experimental time frame. Our study variability was further shifted due to a switch from open air cages to microisolator cages, which is notable, but requires further experimental repeats. These data only include the microisolator-based cage data, in contrast to our previous publication [3]. Additionally, the isolation of our SCFAs involved propanol-based derivatization, which creates propionate and limits any interpretation of that SCFA. Interpretation is limited to the prebiotic and probiotics tested, each of which may act differently in human microbiomes. Lastly, the culture-based resistance profile indicated some redundancy in the picking of colony isolates for each treatment group, potentially skewing the data due to pseudoreplication. Genotypic and phenotypic detection methods both have value in determining the functional levels of antibiotic resistance in the microbiome. However, a more quantitative approach that merges these two types of detection is likely needed for rapid and reliable AMR analysis [44].

In conclusion, this study highlights the potential of prebiotics, specifically inulin-type fructans, to promote gut microbiome recovery after antibiotic-induced dysbiosis. Through comprehensive analyses, we demonstrated that prebiotics significantly altered microbiome metrics compared to probiotics and unmanipulated recovery. Prebiotics restore microbiome diversity, enhance beneficial SCFA production, and inhibit opportunistic bacterial expansion. While probiotics also contributed to pathogen suppression, they delayed microbiome recovery and suppressed overall diversity compared to the prebiotic treatment. Notably, the prebiotic-treated microbiomes exhibited unique culturable bacteria antibiotic resistance profiles, emphasizing the need for further investigation into the safety and long-term implications of prebiotic use. These findings highlight prebiotics’ efficacy in re-establishing microbiome diversity, SCFA production, and call for a nuanced approach to their application. Future studies should integrate both culture-based and genomic methodologies to optimize microbiome diversity and mitigate the risks associated with spreading antibiotic resistance.

## Figures and Tables

**Table 1:** Opportunist bacteria across separate experiments.

Cage Style	Primary Opportunist	Secondary Opportunist
Open Air	*Escherichia coli*	*Shigella* sp.
Open Air	*Escherichia coli*	*Shigella* sp.
Open Air	*Klebsiella oxytoca*	*Shigella* sp.
Microisolator	*Bacteroides thetaiotaomicron*	*Blautia producta*
Microisolator	*Bacteroides thetaiotaomicron*	*Blautia producta*
Microisolator	*Acutalibacter muris*	*Butyricicocus* sp.

## Data Availability

The raw data are available from the NCBI short read archive reference PRJNA1447581.
